# Resilient road safety modeling through spatially disaggregated explainable AI

**DOI:** 10.1371/journal.pone.0344380

**Published:** 2026-04-24

**Authors:** Huashan Ye, Baowen Wu, Dong Yuan, Xue Yang, Qiao Peng, Ruibing Kou

**Affiliations:** 1 School of Artificial Intelligence, Wenshan University, Wenshan, China; 2 Queen’s Business School, Queen’s University Belfast, Belfast, United Kingdom; 3 Shenzhen Research Institute of Shandong University, Shenzhen, China; 4 School of Design and Art, Changsha University of Science and Technology, Changsha, China; University of Salerno: Universita degli Studi di Salerno, ITALY

## Abstract

Understanding spatial disparities in traffic accident severity is essential not only for improving transport safety but also for advancing sustainable and inclusive transportation systems. Road crashes represent a persistent public health and equity challenge, directly linked to the United Nations Sustainable Development Goals (SDG 3: Good Health and Well-Being and SDG 11: Sustainable Cities and Communities). This study proposes an explainable, spatially segmented machine learning framework to examine urban-rural heterogeneity in crash outcomes, using disaggregated accident data from Kent, UK (2022–2024). By treating urban and rural systems as separate analytical units, the study captures risk heterogeneity that conventional pooled approaches often obscure. Among five tested models, Random Forest achieved the best performance and was further interpreted using SHapley Additive exPlanations (SHAP) to uncover how key factors differ in influence across spatial contexts. The results reveal a behavioral risk profile dominating in urban areas and infrastructure-driven risks in rural environments. These findings highlight the need for context-sensitive, evidence-based interventions that ensure transport equity across regions. Additionally, the study contributes to sustainable governance frameworks, enabling spatially adaptive risk mitigation, inclusive policy design and the long-term resilience of transport infrastructures.

## 1. Introduction

Road traffic accidents remain a persistent and systemic challenge to achieving safe, inclusive and resilient transport systems [1]. In the UK, road casualty figures have stagnated over the past decade, despite substantial investments in vehicle safety and infrastructure upgrades. According to the UK Department for Transport (2024), 1,607 fatalities and 29,540 serious injuries were reported in 2024 alone, with no sustained decline in recent trends [2]. These statistics reflect not only a chronic public health concern, but also expose limitations in current road governance strategies to adapt to evolving risks. As transportation networks expand and climate-responsive infrastructure initiatives accelerate, ensuring traffic safety is increasingly recognized as a core dimension of intelligent urban transition and spatially adaptive planning.

A growing body of research highlights the spatial disparities in crash severity between urban and rural environments, an issue that reflects deeper structural imbalances in transport safety systems. Although rural accidents are less frequent, they are significantly more likely to result in fatal or serious injuries due to factors such as higher travel speeds, limited lighting, delayed emergency response and fragmented infrastructure networks [3]. Urban areas, while experiencing more frequent incidents, tend to produce less severe outcomes, reflecting slower traffic, more compact road geometries and stronger enforcement [4]. These contrasting risk landscapes underscore the need for spatially differentiated safety strategies that go beyond aggregate analysis and account for localized infrastructure vulnerability and behavioral dynamics.

This study aims to bridge that gap by proposing a spatially disaggregated, explainable machine learning framework, using separate models for urban and rural environments based on crash data from Kent, UK. Traffic accident severity is shaped by a multitude of interacting factors, including road infrastructure, vehicle maneuvers, lighting conditions and driver attributes, whose relationships are often nonlinear and context-dependent [5]. Traditional statistical models, constrained by linear assumptions, struggle to capture such complexity. Machine learning algorithms offer greater flexibility in modeling these heterogeneous risk mechanisms. When integrated with model interpretability tools such as SHapley Additive exPlanations (SHAP), they enable not only accurate prediction but also transparent, context-aware understanding of key risk contributors [6]. This approach reveals how influential features vary across spatial settings, providing actionable insights for resilience-oriented, place-based traffic safety governance. The study thus contributes methodologically by combining spatial segmentation with model transparency, and practically by informing adaptive interventions aligned with intelligent urban transition.

Empirically, this study is based on the Road Safety Data provided by the UK Department for Transport, focusing on personal injury collisions reported in Kent from January 2022 to November 2024. Kent was selected as the study area due to its unique combination of spatial, infrastructural and operational diversity. The county encompasses a wide range of settlement densities, from compact urban centers to low-traffic rural corridors, enabling the examination of traffic safety disparities under varied contextual conditions. Its role as a strategic transport corridor between London and mainland Europe further introduces complex risk patterns involving high-speed freight, interurban travel and local mobility. These features, along with the high quality and continuity of its crash reporting system, make Kent a methodologically and policy-relevant case for exploring spatially differentiated road safety strategies. After data cleaning and pre-processing, the dataset is split into urban and rural segments based on official spatial classifications. Models are trained using five machine learning techniques, with performance evaluated through accuracy, precision, recall and F1-score metrics. The best-performing model is further interpreted using SHAP to extract actionable knowledge regarding the determinants of crash severity in different contexts. Through linking predictive analytics with interpretable outputs, the study offers actionable insights for spatially adaptive road safety strategies and resilience-oriented transport governance.

This work makes three key contributions. First, a spatially disaggregated analytical framework that explicitly separates urban and rural traffic systems is proposed, moving beyond conventional pooled modeling approaches that mask spatial heterogeneity in accident severity mechanisms. Second, by integrating Random Forest models with SHAP-based interpretation, the study provides a comparative, explainable analysis of how the importance and influence of risk factors differ across spatial contexts, rather than merely explaining a single global model. Third, the study translates these context-specific insights into governance- and equity-relevant evidence, demonstrating how interpretable machine learning can support spatially adaptive, data-driven road safety strategies aligned with sustainable and resilient transport planning.

The remainder of this paper is structured as follows. Section 2 reviews the relevant literature on accident severity prediction, machine learning applications in transport safety and spatial disparities between urban and rural crash outcomes. Section 3 introduces the data source and variable selection. Section 4 presents the modeling methodology. Section 5 discusses empirical results, including model comparison, SHAP-based interpretation and policy implications for road safety. Finally, Section 6 concludes the paper and outlines future research directions.

## 2. Literature review

### 2.1 Key factors influencing accident severity

Research on the determinants of traffic accident severity has evolved significantly over the past two decades, with increasing emphasis on contextual, spatial and behavioral dimensions. A wide range of factors have been identified, spanning road geometry, environmental conditions, driver behavior, vehicle characteristics and temporal patterns.

Among the earliest recognized factors, weather and visibility have received substantial attention. A systematic review by Theofilatos and Yannis (2014) found that while precipitation generally increases accident frequency, its effect on severity remains unclear. The influence of visibility and other weather factors on crash severity was found to be inconsistent, suggesting the need for further investigation using real-time environmental data [7]. Gao et al. (2020) found that under hazy weather, time-to-collision indicators increased by 35.9% (time exposed time) and 43.0% (time integrated time), pointing to higher crash risk and reduced car-following ability under limited visibility [8].

Speed limits, on the other hand, exhibit a more consistent relationship with severity. Lee et al. (2006) developed a real-time crash prediction model using microsimulation and found that implementing variable speed limits could reduce crash potential by 5–17%. The study highlighted the role of dynamic control strategies such as adjusting intervention thresholds and speed change magnitudes in mitigating risks under unstable traffic conditions [9]. More recently, Hasan et al. (2023) demonstrated that variable speed limit and advisory speed systems reduced crash frequency by 15.97% and 26.14%, respectively, using high-resolution short-term traffic models [10].

Junction characteristics also play a vital role. Wu et al. (2019) analyzed over 4,000 urban junctions in the UK and found that crash severity patterns vary significantly across different junction type. While roundabouts and mini roundabouts exhibited the lowest casualty rates, T-junctions, staggered junctions and crossroads were linked to the highest crash risks, particularly in the absence of signals or pedestrian facilities [11]. Using a neural network model, Siamidoudaran et al. (2019) similarly identified T-shaped and staggered junctions as injury severity hotspots in central London, further emphasizing the critical roles of signal control, crossing infrastructure and peak-hour traffic in aggravating crash outcomes [12].

Temporal variables, such as time of day and weekly patterns, have also been shown to influence accident severity. Alhaek et al. (2024) highlighted the importance of temporal variables in traffic accident severity prediction, demonstrating that incorporating time-dependent features via Bidirectional Long Short-Term Memory networks significantly improved model performance. Their findings suggest that accident severity is dynamically influenced by temporal patterns, underscoring the need to integrate such variables in predictive modeling [13].

### 2.2 Evolution of modelling approaches

Traditional statistical methods formed the foundation of early accident research. Zong et al. (2013) compared Bayesian networks and regression models for predicting accident severity across fatality, injury and property damage indicators, and found that Bayesian models outperformed other models in both model fit and prediction accuracy [14]. Xi et al. (2014) applied logistic regression (LR) to model crash severity on curved road segments in China, validating their model using maximum likelihood estimation and Hosmer-Lemeshow tests [15]. Similarly, Delen et al. (2006) developed a series of artificial neural networks to capture nonlinear relationships between crash-related factors and injury severity levels [16]. While these models improved sensitivity to complex variable interactions, their effectiveness often depended on access to large datasets and intensive training to ensure generalizability.

The rise of machine learning methods has addressed many of these limitations. Techniques such as convolutional neural network (CNN), support vector machines (SVM) and Random Forests (RF) have been widely applied to crash severity prediction tasks. Rahim and Hassan (2021) proposed a deep learning framework using CNNs and customized loss function to predict crash severity in highway work zones, demonstrating improved precision and recall for fatal and injury crashes [17]. Similarly, Manzoor et al. (2021) proposed an ensemble model that fuses RF and CNN at the decision level to identify key predictors and improve classification accuracy, achieving an F-score of 0.980 on U.S. crash records [18]. Yang et al. (2022) proposed a multi-task deep neural network framework to simultaneously predict multiple types of crash severity, including injury, death and property loss, integrating layer-wise relevance propagation to enhance model explainability and identify key contributing factors [19]. Kitali et al. (2021) employed a SVM model trained by the Firefly Algorithm to identify key factors contributing to crash injury severity on express lane facilities, leveraging crash data from California to model geometric and environmental risk influences [20].

Machine learning methods have also been expanded to incorporate spatial features. Yang et al. (2022) developed an accident analysis framework based on the geographic information system, integrating spatial partitioning and kernel density estimation with machine learning techniques to assess maritime accident severity across the Fujian Sea area. They compared RF, Adaboost, gradient boosting decision tree and stacking models to characterize spatial risk distribution using autocorrelation measures [21].

One of the key methodological challenges in severity prediction is class imbalance. Since fatal and serious accidents are rare relative to slight ones, predictive models tend to favor the majority class, resulting in disappointing performance for high-severity cases. To address this, Li et al. (2023) proposed a deep learning approach that improves class balance by generating additional samples for severe crash cases. Combined with XGBoost, the method enhanced prediction performance and used SHAP to identify key factors behind injury severity [22]. Formosa et al. (2023) systematically evaluated the effectiveness of several machine and deep learning models under class-imbalanced traffic conflict datasets. Their findings highlighted the superior performance of deep neural networks in real-time conflict prediction, particularly in sensitivity and reliability under skewed class distributions [23].

Recent advances in spatial safety modeling have further extended machine learning approaches by explicitly incorporating spatial dependence and multi-scale risk structures. Abdulrazaq and Fan (2025) propose a spatial safety modeling framework that integrates priority-based classification with spatial analytics to capture heterogeneity in crash risk patterns. By explicitly modeling spatial dependence, their results show that geographically differentiated severity determinants can be identified, enhancing both interpretability and policy relevance [24].

### 2.3 Urban-rural comparison: gaps and opportunities

Despite extensive literature on crash prediction, direct comparisons between urban and rural traffic contexts remain underexplored, especially in the UK. Most models are trained on pooled data, which masks the distinct risk profiles and intervention levers of different road environments.

Champpahom et al. (2023) examined urban-rural disparities in motorcycle crash severity using recursive probabilistic logic model with variables models on Thai local road data, but focused narrowly on young adult riders and did not incorporate machine learning techniques or broader policy variables, limiting its generalizability [4]. Dong et al. (2022) applied SHAP-enhanced boosting models to highway crash data in Pakistan, identifying driver age, lighting condition and collision type as dominant predictors of severity outcomes. The identified features such as pedestrian involvement and trailer impacts are often spatially clustered and exhibit distinct distributions across road environments, offering transferable insight for urban and rural disaggregation studies [25]. Abdulrazaq and Fan (2025) employ temporally disaggregated injury severity models to examine how the influence of key explanatory variables varies across time. Their results show that temporally segmented models uncover systematic variation in severity determinants that is obscured in aggregated analyses, particularly for high-severity outcomes. These findings highlight the value of context-sensitive and interpretable modeling frameworks for informing more targeted and robust safety interventions [26].

More recent efforts advocate spatially aware, interpretable machine learning frameworks to inform regional safety strategies. Park and Hong (2022) applied deep learning to urban accident risk prediction in Seoul by incorporating both static and dynamic road features such as speed limits, traffic volume and solar angle. Their model achieved 75% accuracy and 81% recall and further enabled real-time risk classification and guidance via a mobile system [27]. Similarly, Gilani et al. (2021) combined logit regression with artificial neural network to analyze crash severity in Rasht city, identifying lighting and road surface quality as critical predictors. Their findings reinforce the relevance of spatially contextualized machine learning models for high-resolution urban risk assessment [28].

Building on these methodological advances and acknowledging the spatial heterogeneity identified in recent research, this study addresses the underexplored intersection of explainable machine learning and urban-rural crash severity analysis. By employing SHAP-enhanced RF models on disaggregated data from Kent, UK, it moves beyond pooled analysis to reveal context-specific determinants of crash severity. In contrast to studies that prioritize predictive performance alone, our approach highlights why certain features matter in different spatial contexts, informing more nuanced interventions. Moreover, this contributes to the growing need for data-driven, equitable, and spatially targeted transport safety strategies that align with the principles of sustainable mobility governance, including SDG 11 (sustainable cities and communities) and Vision Zero road safety frameworks. In doing so, the study not only bridges a methodological gap but also provides an operational foundation for scalable, location-sensitive traffic policy design.

## 3. Data and variables

### 3.1 Data collection

The data used in this study is sourced from the UK Department for Transport’s Road Safety Data, which provides detailed records of road traffic accidents involving personal injury across Great Britain. The dataset includes comprehensive information on accident circumstances, vehicle types involved, road conditions and the severity of casualties.

For the purpose of this study, we extract a subset of data specific to Kent, a county located in Southeast England. Kent was selected due to its well-documented heterogeneity in road typologies, encompassing high-speed A-roads, dense urban street networks and sparsely trafficked rural lanes. This structural diversity provides a representative microcosm of the broader UK road system, making it an ideal case for studying spatial disparities in traffic accident severity. Furthermore, the Kent police jurisdiction has a reliable and consistent accident reporting system, ensuring high data quality with minimal missing values across years. The county has also been the subject of previous road safety studies [29], supporting the generalizability and policy relevance of our findings.

To ensure that the findings are not biased by pandemic-induced travel restrictions, we use accident data from January 2022 to November 2024, a period during which mobility patterns in the UK had returned to post-COVID-19 normality. This timeframe captures current road usage behaviors under stable societal conditions, providing a robust empirical basis for sustainable transportation safety assessment.

A geographically filtered subset corresponding to Kent was extracted from the national dataset, and then further divided into urban and rural segments based on official location classifications provided in the source. The variables selected for analysis are presented in Table 1. These features were chosen based on their theoretical relevance to traffic accident severity, as established in existing literature, and their data quality within the Kent subset. Specifically, we excluded variables with more than 30% missing values to ensure the robustness of subsequent analysis [30]. After this screening process, a total of 31 variables were retained for model development and interpretation.

**Table 1:** Variable list.

Variables	Description
accident_severity	Target Variable
accident_year	
longitude	
Latitude	
date	
day_of_week	
time	
road_type	roundabout, one-way street et al.
speed_limit	valid speed limits: 20, 30, 40, 50, 60, 70
junction_detail	roundabout, crossroads et al.
junction_control	authorized person, auto traffic signal et al.
pedestrian_crossing_human_control	control by school crossing patrol et al.
pedestrian_crossing_physical_facilities	zebra, central refuge et al.
light_conditions	daylight, darkness – lights lit et al.
weather_conditions	fog or mist et al.
road_surface_conditions	wet or damp et al.
special_conditions_at_site	auto traffic signal – out et al.
carriageway_hazards	vehicle load on road et al.
urban_or_rural_area	
vehicle_type	taxi/private hire car et al.
vehicle_manoeuvre	u-turn, changing lane et al.
vehicle_location_restricted_lane	bus lane, footway et al.
junction_location	entering from slip road et al.
vehicle_leaving_carriageway	nearside, offside et al.
vehicle_left_hand_drive	yes or no
sex_of_driver	
age_of_driver	
age_band_of_driver	
engine_capacity_cc	
propulsion_code	Electric, gas et al.
age_of_vehicle	

Additionally, preliminary correlation analysis and variance inflation diagnostics were conducted to assess potential multicollinearity among predictors. While moderate correlations were observed between certain road environment variables, no extreme multicollinearity was identified that would compromise model stability. Given that the primary modeling approach relies on tree-based ensemble methods, which are known to be robust to correlated features, no further variable elimination was applied on this basis.

### 3.2 Data pre-processing

To ensure data quality and analytical consistency, a series of preprocessing steps were applied prior to model development. First, rows containing missing or unknown values were removed, provided that such records accounted for less than 3% of the entire dataset. These missing entries were primarily associated with administrative or auxiliary attributes rather than core outcome or explanatory variables. Given the small proportion of missingness, listwise deletion was adopted as a conservative preprocessing strategy to avoid introducing additional assumptions through imputation. A comparison of key variable distributions before and after removal confirmed that this step did not materially alter the overall data structure. Next, feature extraction and variable transformation were conducted to enhance model interpretability. The hour component was extracted from the original time field to capture diurnal traffic patterns. A new binary variable, is_dst, was created to indicate whether the accident occurred during Daylight Saving Time (DST), following European convention, starting on the final Sunday of March and ending on the last Sunday of October. This derivation was based on the recorded date of each accident.

To prepare the dataset for machine learning algorithms, all variables were processed according to their data types. Categorical variables with more than two categories (e.g., road_type, junction_detail, light_conditions and weather_conditions) were encoded using one-hot encoding, allowing the models to capture non-ordinal category information without imposing artificial numerical relationships. Binary variables, such as is_dst and vehicle_left_hand_drive, were encoded as 0 or 1 for model consistency. Continuous variables were retained in their original numerical form. Tree-based models are inherently robust to high-dimensional sparse feature representations generated by one-hot encoding, while the same encoded feature set was consistently used across all models to ensure comparability.

Additionally, outliers in continuous variables, such as engine_capacity_cc, age_of_driver and age_of_vehicle, were identified using interquartile range thresholds. Values far outside the normal range were either removed or capped if they were clearly erroneous (e.g., driver age < 0 or engine capacity > 20,000cc). This step mitigates the influence of extreme values that could distort model training outcomes while preserving valid variation.

After cleaning and transformation, descriptive statistics of the variables used for model development in urban and rural settings, together with detailed explanations of variable codes and category definitions, are reported in Appendix A.1 (Tables A1 and A2). The comparison reveals several consistent patterns across both spatial contexts. In both urban and rural areas, most accidents occurred on Fridays, typically during daylight hours, and predominantly on single carriageway roads. These accidents frequently involved male drivers, with the majority of vehicles being passenger cars, followed by light commercial vans. Environmental conditions were generally favorable, with fine weather and dry road surfaces being the most commonly recorded conditions. This finding is consistent with existing literature suggesting that accident frequency is influenced not only by road hazards but also by exposure and driver behavior. Specifically, favorable conditions typically lead to increased travel activity and potentially lower risk perception, contributing to a higher number of total accidents. Conversely, during poor weather conditions, drivers often reduce speed, drive more cautiously, or avoid travel altogether, thereby mitigating risk. These interpretations align with findings by Quddus (2008), who observed that a large proportion of accidents in London occurred under clear weather conditions due to increased road usage [31]. Similarly, Theofilatos and Yannis (2014) concluded that while per-trip accident risk may rise in poor weather, the overall volume of accidents is often greater during fine and dry conditions due to higher exposure [7].

Besides, most accidents occurred away from junctions, and when junctions were involved, T-junctions, roundabouts and crossroads were among the most frequent types depending on the area. Manual or uncontrolled junctions and absence of pedestrian crossing controls were also prominent, suggesting potential infrastructure gaps. Additionally, drivers involved were typically in the 26–45 age range, and the average vehicle age was around 6 years in both settings.

Notably, while the patterns between urban and rural areas are broadly similar, several meaningful differences emerge. Urban areas showed a higher proportion of accidents at roundabouts and lower speed limits (30–40 mph), whereas rural accidents were more frequently associated with crossroads, higher speeds and larger open road segments. Vehicle engine capacities in urban areas were slightly higher on average, while rural accidents involved more pedal cycles and incidents occurring on footways or hard shoulders.

### 3.3 Data exploration and visualization

To gain preliminary insights into spatial and behavioral factors influencing accident severity, the Kent dataset was partitioned into urban (n = 13,616) and rural (n = 13,246) subsets. A series of visual explorations were conducted to compare variable distributions and accident patterns across different geographical contexts.

**Fig 1:**
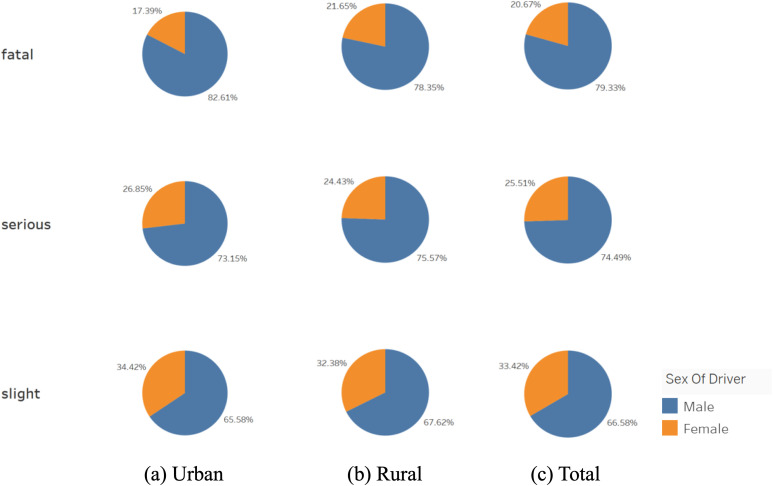
Relationship between posted speed limits and accident severity.

Fig 1 investigates the gender distribution of drivers across different levels of accident severity. In both urban and rural contexts, male drivers consistently represent a larger proportion of more severe accidents. This trend becomes especially pronounced in fatal crashes, where the male share exceeds 80% in urban areas. Such a disparity suggests underlying behavioral differences in driving patterns. A prior study conducted by Rhodes and Pivik (2011) have shown that male drivers are more prone to risk-taking and aggressive behaviors, particularly in complex traffic scenarios or high-speed environments, which significantly elevates their likelihood of involvement in serious or fatal collisions [32].

**Fig 2:**
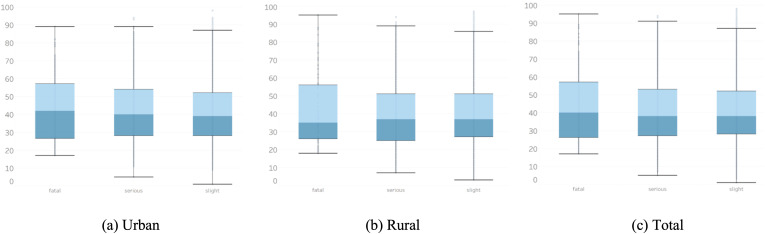
Age distribution of drivers.

Fig 2 presents the age distribution of drivers involved in accidents. While slight and serious accidents show similar age profiles across regions, fatal accidents in rural areas exhibit a significantly lower median age. This indicates a greater vulnerability among younger drivers in rural settings, possibly linked to inexperience, limited access to emergency response or poor road infrastructure. Interestingly, the upper age range (upper hinge) remains comparable, indicating that the observed disparity is driven by a left-skewed distribution, with more young drivers involved in fatal accidents, rather than an overall expansion of the age range.

## 4. Methodology

Recent work has demonstrated that integrating machine learning with spatial segmentation techniques can effectively capture the heterogeneous risk profiles of road environments under evolving mobility transitions [33]. To model and compare accident severity across urban and rural road environments, we implemented a series of supervised machine learning classification algorithms. These methods were selected to represent a diverse set of modeling paradigms in terms of linearity, interpretability and learning complexity. The target variable is accident_severity, which includes three classes: slight, serious and fatal.

Prior to model training, the dataset was randomly partitioned into a training set (70%) and a test set (30%) to enable out-of-sample performance evaluation. The training set was used for model fitting, hyperparameter tuning, and oversampling procedures, while the test set was held out and remained untouched throughout model development. To further ensure robustness and mitigate variance in model estimation, stratified five-fold cross-validation was applied within the training data, preserving the original class distribution across folds.

The models used in this study include: LR, Support Vector Classifier (SVC), Decision Tree (DT), RF and Gradient Boosted Trees (GBT). Among all models, RF exhibited the best overall performance and was therefore selected for subsequent in-depth analysis. To address class imbalance, particularly the underrepresentation of serious and fatal accidents, we compared three oversampling techniques: Random Oversampling, Adaptive Synthetic Sampling (ADASYN) and BorderlineSMOTE. After empirical comparison based on validation performance, BorderlineSMOTE was selected. All oversampling procedures were applied exclusively to the training data, while validation and test sets retained the original class distribution, in order to prevent information leakage and reduce the risk of overfitting. Models were trained and evaluated using stratified 5-fold cross-validation, ensuring that each fold retained the original class proportions. Given the multi-class nature of accident severity prediction and the presence of class imbalance, model performance was evaluated using multiple complementary metrics rather than relying on accuracy alone. Specifically, accuracy, precision, recall, and F1-score were used to assess classification performance across severity levels. Precision and recall capture different aspects of classification error, while the F1-score provides a balanced measure when class distributions are uneven. This multi-metric evaluation strategy is widely adopted in transport-related classification studies to ensure a robust and reliable assessment of model performance [34].

Given the pronounced class imbalance in injury severity data, model performance was evaluated not only using overall accuracy but also using per-class precision, recall, and F1-scores. In addition, normalized confusion matrices were employed to examine misclassification patterns across severity levels and to assess the model’s ability to distinguish high-severity outcomes, particularly serious and fatal crashes. The model with the best trade-off across these metrics was selected for interpretation analysis.

### 4.1 Oversampling methods

In multi-class classification problems, class imbalance can significantly hinder the model’s ability to correctly identify underrepresented classes, particularly in achieving high recall rates for minority categories. To mitigate this issue, this study evaluates and applies oversampling techniques aimed at improving the distribution of samples in the training dataset.

One widely used approach is the Synthetic Minority Oversampling Technique (SMOTE), a heuristic method that generates new synthetic samples by interpolating between existing minority instances and their nearest neighbors. This not only increases the representation of minority classes but also avoids the overfitting commonly associated with simple random oversampling methods. Formally, given a minority class sample $x_{i}$ and one of its k-nearest neighbors $x_{j}$, a synthetic sample $x_{n}$ is generated using the following equation:


$$\begin{array}{c} x_{n}=x_{i}+rand(\text{0},\text{1})\times \left|x_{i}-x_{j}\right| \end{array}$$ (1)

This process is repeated until the number of minority class samples matches that of the majority class, resulting in a more balanced training distribution. Building upon this, we employ Borderline-SMOTE, an enhanced version of SMOTE that targets samples located near the decision boundaries, areas where misclassification risk is highest. Instead of oversampling all minority instances indiscriminately, Borderline-SMOTE first evaluates each minority sample by identifying its m nearest neighbors from the full dataset and counts how many $m^{\prime }$ belong to other classes. Based on this ratio, samples are classified as follows: 1) Noise: if all m neighbours belong to other classes ($m^{\prime }=m$); these are excluded from synthesis; 2) Danger: if over half but not all of the mmm neighbors belong to other classes ($m/\text{2}\leq m^{\prime }<m$); these are considered borderline cases and are selected for oversampling; and 3) Safe: if most neighbors are of the same class ($m^{\prime }<m/\text{2}$); these are not oversampled. New synthetic samples are then generated only from the Danger category using the following interpolation:


$$\begin{array}{c} x_{n}=x_{i}+\alpha \left(x_{zi}-x_{i}\right) \end{array}$$ (2)

where $x_{zi}$ is a randomly selected neighbor of the same class and α∈(0,1) is a random coefficient [5].

In total, three oversampling strategies were tested in this study: Random Oversampling, Borderline-SMOTE and ADASYN. These methods were compared using RF models, with their performance evaluated through cross-validated classification accuracy. As reported in Table 2, Borderline-SMOTE demonstrated the most consistent and superior performance, and was therefore selected for final model training.

**Table 2:** Oversampling methods comparation (Test Set).

	Urban	Rural
Borderline-SMOTE	93.0%	92.5%
Random Oversampling	87.9%	85.4%
ADASYN	76.5%	80.3%

### 4.2 Machine learning models

LR was employed as a baseline linear classification model. Its probabilistic formulation and interpretability make it a commonly used reference method in accident severity analysis, particularly for assessing the marginal effects of explanatory variables. However, its reliance on linear decision boundaries limits its ability to capture complex non-linear interactions among risk factors.

SVC is a kernel-based learning method that constructs optimal separating hyperplanes in a transformed feature space. By utilizing non-linear kernel functions, SVC can model more complex decision boundaries than linear models, making it suitable for high-dimensional classification tasks. Nevertheless, its performance can be sensitive to kernel choice and parameter tuning, especially in imbalanced multi-class settings.

DT is a rule-based classification method that recursively partitions the feature space based on impurity reduction criteria. Its transparent structure allows intuitive interpretation of decision paths, which is valuable for policy-oriented analysis. However, single-tree models are prone to overfitting and instability, motivating the comparison with ensemble-based approaches.

GBT is an ensemble learning technique that builds a sequence of decision trees in a stage-wise manner, where each tree corrects the residual errors of the previous ones. This boosting strategy enables GBT to capture complex non-linear relationships and interactions among variables, often achieving strong predictive performance in transport-related classification tasks. Recent studies have demonstrated that ensemble tree-based models, including both Random Forest and boosting-based approaches, are particularly effective for multi-class transport prediction problems involving heterogeneous inputs and class imbalance [35].

Among the classification models evaluated in this study, RF emerged as the most effective in handling the multi-class accident severity prediction task. RF is an ensemble learning algorithm based on DT, designed to improve predictive performance through the combination of multiple weak learners.

Each decision tree in the forest is trained on a bootstrap sample (i.e., sampling with replacement) from the training dataset. At every node split, only a random subset of features is considered. This process introduces both data diversity and feature randomness, which together reduce variance and mitigate overfitting, an issue common in single-tree models.

Formally, given a training dataset $D=\overset{n}{\underset{i=\text{1}}{\left\{\left(x_{i},y_{i}\right)\right\}}}$, where $x_{i}\in R^{d}$ is the feature vector and $y_{i}$ is the class label, RF builds B decision trees $\overset{B}{\underset{b=\text{1}}{\left\{h_{b}(x)\right\}}}$, each trained on a bootstrapped dataset $D_{b}\subset D$. The final prediction $\hat{y}$ for an input sample x is obtained by majority voting across all trees:


$$\begin{array}{c} \hat{y}=mode\left(\overset{B}{\underset{b=\text{1}}{\left\{h_{b}(x)\right\}}}\right) \end{array}$$ (3)

Each individual tree $h_{b}$ recursively partitions the feature space by selecting the feature and split point that maximize the reduction in impurity. For classification tasks, impurity is commonly measured using the Gini index:


$$\begin{array}{c} G(t)=\text{1}-\sum_{k=\text{1}}^{K}\overset{\text{2}}{\underset{k}{p}} \end{array}$$ (4)

where $p_{k}$ is the proportion of samples of class k in node t, and K is the total number of classes. The best split is chosen to maximize the decrease in Gini impurity after partitioning.

An additional advantage of RF is its ability to compute feature importance, typically based on the average reduction in impurity attributed to each feature across all trees:


$$\begin{array}{c} FI(f)=\frac{\text{1}}{B}\sum_{b=\text{1}}^{B}\sum_{t\in T_{b}}△G_{t}(f) \end{array}$$ (5)

where $△G_{t}(f)$ is the reduction in Gini impurity from splitting node t using feature f, and $T_{b}$ is the set of nodes in tree b [36].

### 4.3 Model interpretation

To interpret the predictions of non-linear machine learning models and to uncover the relationships between input features and accident severity outcomes, this study employs SHAP. SHAP is an explainable artificial intelligence technique grounded in cooperative game theory that quantifies the contribution of each input variable to individual model predictions. By providing both global and local explanations, SHAP enables transparent interpretation of complex, non-linear input-output relationships that are otherwise difficult to extract from ensemble models. Such interpretability is increasingly recognized as essential for ensuring transparency, accountability, and policy relevance in applied machine learning studies [37].

SHAP i attributes each feature an importance value for a particular prediction [38]. The SHAP value $\emptyset_{j}$ for feature j is computed as follows:


$$\begin{array}{c} \emptyset_{\text{j}}=\sum_{S\subseteq \text{N}\backslash \left\{\text{j}\right\}}\;\frac{|S|!\left(|N|-|S|-\text{1}\right)!}{|N|!}\left(\text{v}\left(S\cup \left\{\text{j}\right\}\right)-\text{v}(S)\right) \end{array}$$ (6)

where N is the set of all features, S is a subset of N excluding j, and v(S) is the model output based on features in S. This reflects the marginal contribution of feature j averaged over all possible feature subsets.

SHAP values can be integrated into a linear explanation model as below:


$$\begin{array}{c} g\left(z^{\prime }\right)=\varphi_{\text{0}}+\sum_{j=\text{1}}^{M}\;\varphi_{j}\overset{\prime }{\underset{j}{z}} \end{array}$$ (7)

where $g\left(z^{\prime }\right)$ is the approximated model output, $\overset{\prime }{\underset{j}{z}}$ indicates the presence of feature j, and $\varphi_{j}$ is the contribution of that feature. SHAP thus provides global interpretability and local explainability, supporting transparency and trust in model predictions [39].

SHAP visualizations provide an intuitive graphical representation of the relationship between input features and model outputs by quantifying both the magnitude and direction of each variable’s contribution to predicted accident severity.

## 5. Results and discussion

### 5.1. Model performance comparison

Table 3 presents a comparative evaluation of the five machine learning models (LR, SVC, DT, RF and GBT) applied separately to the urban and rural subsets. Four commonly used metrics are reported for each model: accuracy, precision, recall, and F1-score, offering a holistic view of classification performance across both balanced and imbalanced outcomes.

**Table 3:** Machine learning models comparation.

	Urban				Rural			
	Accuracy	Precision	Recall	F1-score	Accuracy	Precision	Recall	F1-score
LR	62.1%	0.62	0.62	0.61	60.8%	0.60	0.61	0.60
SVC	67.4%	0.68	0.67	0.64	62.1%	0.63	0.62	0.59
DT	89.4%	0.90	0.89	0.89	89.1%	0.89	0.89	0.89
RF	93.0%	0.93	0.93	0.93	92.5%	0.92	0.92	0.92
GBT	78.0%	0.77	0.78	0.77	75.9%	0.75	0.76	0.75

Among all models, RF consistently achieves the highest scores across all evaluation metrics for both urban and rural datasets. Specifically, it achieves 93.0% accuracy and an F1-score of 0.93 on the urban subset, and 92.5% accuracy with an F1-score of 0.92 on the rural subset. In contrast, LR and SVC perform notably worse, especially on the rural data, likely due to their limited ability to capture complex, non-linear relationships. While the DT model performs reasonably well, its single-tree structure may be prone to overfitting, which the ensemble-based RF mitigates through variance reduction. GBT also shows strong results but slightly underperforms compared to RF and involves greater computational cost and hyperparameter sensitivity.

Based on this comprehensive evaluation, RF is selected as the final model for sub-sequent interpretability analysis and spatial comparison of variable influence, as it provides the most balanced and reliable predictive performance across both urban and rural traffic environments.

Beyond overall accuracy, per-class performance metrics also indicate that the Random Forest model achieves high precision and recall for slight crashes, while maintaining meaningful discriminatory power for serious and fatal outcomes. Normalized confusion matrices (Appendix B) show that misclassification of high-severity crashes primarily occurs between fatal and serious categories, rather than being incorrectly predicted as slight. This pattern is consistent across urban and rural settings and provides evidence that the model meaningfully differentiates high-severity crashes without defaulting to the majority slight category.

### 5.2 Urban feature interpretation

**Fig 3:**
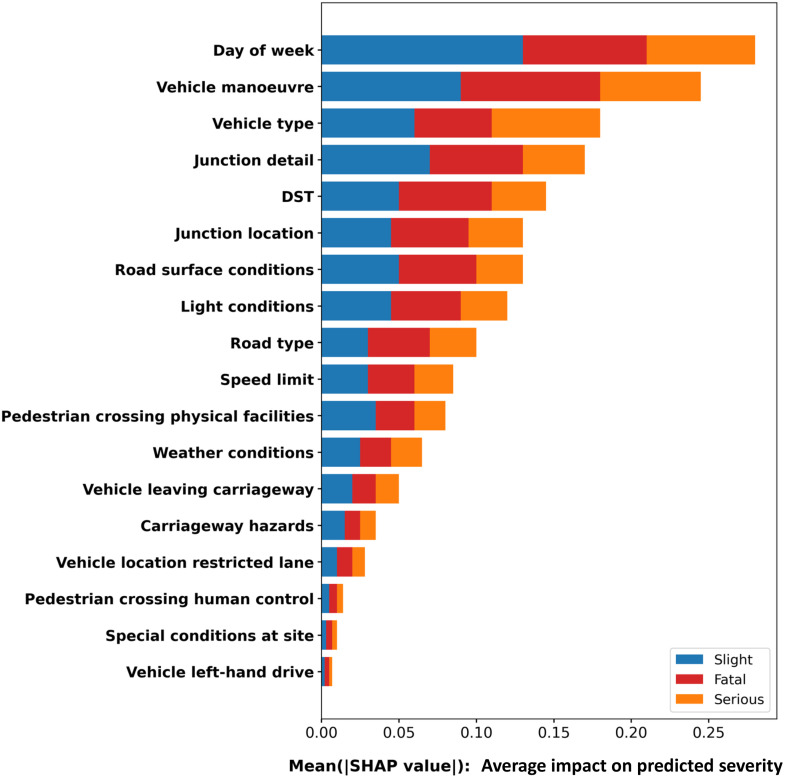
SHAP feature importance summary plot (urban).

To better understand the drivers of traffic accident severity in urban settings, we utilized SHAP to interpret the output of the RF classifier. As shown in Fig 3, the five most influential variables contributing to severity prediction in urban areas include day of the week, vehicle maneuver, vehicle type, junction detail and daylight-saving time (is_dst).

**Fig 4:**
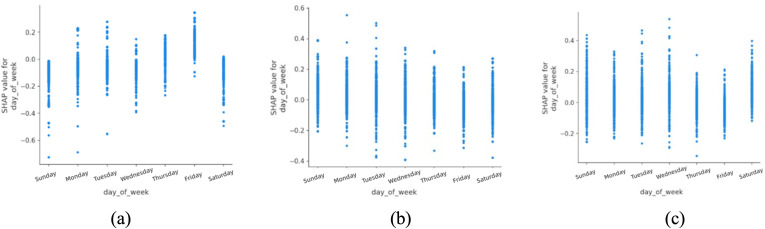
SHAP partial dependency plot for day_of_week (urban).

The analysis of day of the week (Fig 4) indicates that fatal accidents are more frequently associated with Fridays in urban areas. This pattern may reflect differences in exposure and travel composition toward the end of the workweek, such as increased traffic volume, longer trip distances, or a higher proportion of discretionary travel. In contrast, non-fatal accidents appear more evenly distributed across weekends, which may be influenced by reduced commuting pressure and altered enforcement or travel behaviors. These associations suggest that temporal variation in traffic demand and user composition should be considered when designing adaptive traffic management and public transport strategies, rather than implying a direct causal effect of specific weekdays.

**Fig 5:**
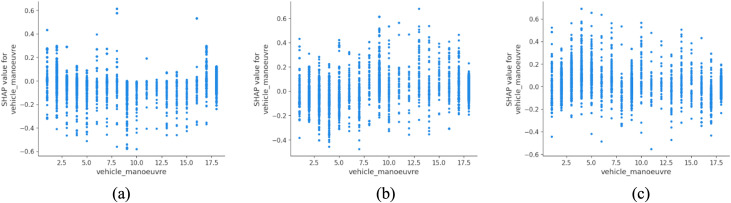
SHAP partial dependency plot for vehicle_manoeuvre (urban).

Vehicle maneuver types also exhibit a strong association with severity outcomes (Fig 5). Right-turning vehicles, particularly those waiting or initiating a turn, are more frequently associated with slight and serious injuries. In left-hand traffic systems such as the UK, these maneuvers typically involve complex interactions across multiple lanes and limited visibility, which may contribute to higher observed injury severity. Similar associations have been reported by Ukkusuri et al. (2020), who identified turn-related crashes as a key contributor to urban injury patterns in multimodal environments [40].

**Fig 6:**
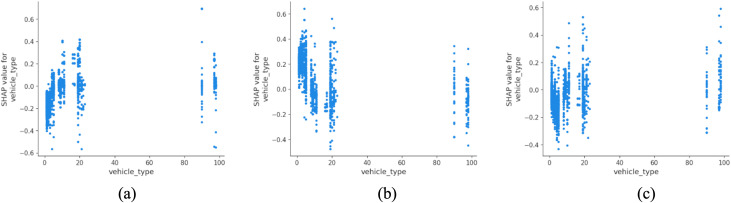
SHAP partial dependency plot for vehicle_type (urban).

Vehicle type plays an equally critical role (Fig 6). While larger vehicles such as buses and coaches are more strongly associated with fatal outcomes, two-wheeled motorized vehicles (e.g., scooters and motorcycles) show the highest proportion of serious injuries. This bimodal pattern may reflect differences in vehicle mass, impact dynamics, and user protection levels, rather than implying a direct causal relationship. These findings are consistent with work by Santos et al. (2021), who reported higher injury severity among powered two-wheeler users in European urban areas [41]. A shift toward sustainable urban transport must therefore also address safety equity, including investment in protected lanes, stricter licensing and education for micro-mobility users.

**Fig 7:**
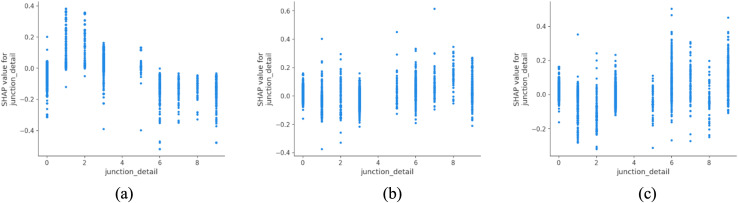
SHAP partial dependency plot for junction_detail (urban).

The analysis of junction types (Fig 7) shows that roundabouts and mini roundabouts are more frequently associated with fatal outcomes in the model predictions. This pattern does not imply that roundabouts inherently increase crash severity, but may reflect contextual factors such as traffic volume, vehicle mix, visibility constraints, and driver behavior in complex urban junction environments. In contrast, simpler configurations such as crossroads and private entrances are more often associated with lower-severity outcomes, potentially due to lower operating speeds and clearer sightlines. These associations highlight the importance of context-sensitive intersection assessment that accounts for exposure and design characteristics, rather than attributing severity outcomes to junction type alone [42].

**Fig 8:**
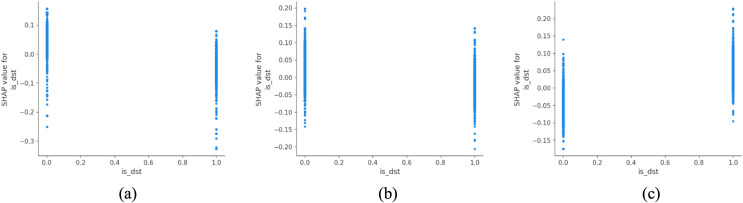
SHAP partial dependency plot for is_dst (urban).

Finally, daylight-saving time (DST) is associated with a lower predicted probability of fatal outcomes, while slight accidents become relatively more common (Fig 8). This association may be influenced by changes in visibility, travel timing, and exposure patterns during extended daylight hours, rather than a direct protective effect of daylight alone. Accordingly, temporal lighting and enforcement strategies should be interpreted as complementary measures that interact with broader travel behavior and environmental conditions.

### 5.3 Rural feature interpretation

**Fig 9:**
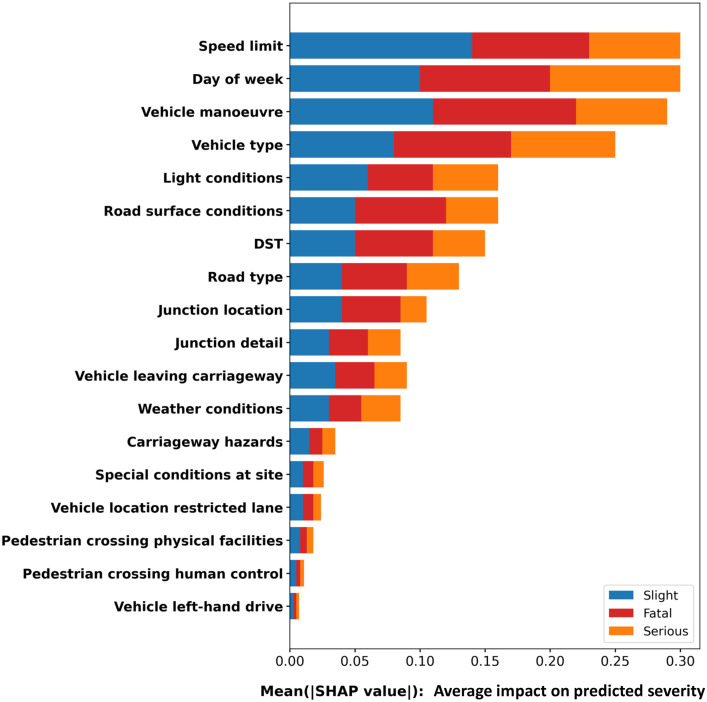
SHAP feature importance summary plot (rural).

To further explore the heterogeneity in traffic accident severity between spatial contexts, we analyzed key predictors of severity outcomes in rural areas using SHAP-based interpretation of the RF model. Fig 9 presents the feature importance ranking, identifying speed limit, day of the week, vehicle maneuver, vehicle type and light conditions as the five most influential variables. Compared with urban areas, these rural predictors reflect a stronger interaction with environmental and infrastructural risk factors, aligning with a prior study conducted by Ziakopoulos and Yannis (2020) that emphasized the distinct challenges of rural road safety [43].

**Fig 10:**
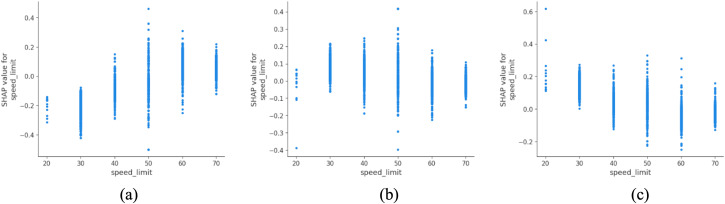
SHAP partial dependency plot for speed_limit (rural).

The posted speed limit (Fig 10) shows a strong association with predicted accident severity in rural areas. Higher speed limits are associated with a greater proportion of fatal outcomes and a lower proportion of slight injuries in the model predictions. This pattern may reflect a combination of higher operating speeds, road design standards, and traffic composition typically observed on rural high-speed corridors, rather than the effect of speed limits in isolation. These findings are consistent with Van (2015), who reported elevated severity levels on higher-speed rural roads [44]. From a policy perspective, this underscores the importance of context-sensitive speed management as part of a sustainable rural mobility strategy, where enforcement capacity is limited and infrastructure may not support high-speed travel safely.

**Fig 11:**
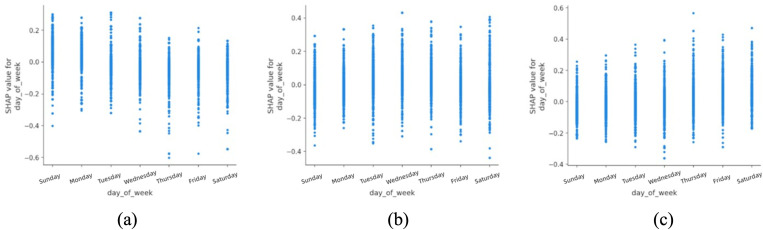
SHAP partial dependency plot for day_of_week (rural).

Day of the week (Fig 11) was found to have a relatively minor impact in rural areas compared to urban zones. This difference may stem from the fact that rural travel is less tightly coupled to weekday commuting patterns and more influenced by agricultural, leisure or long-distance travel.

**Fig 12:**
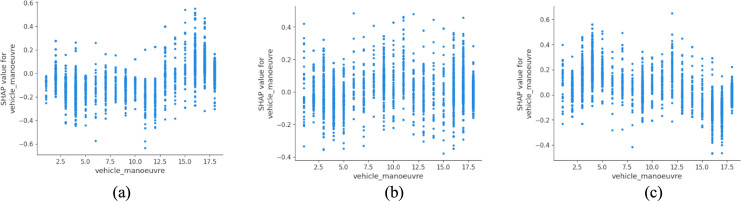
SHAP partial dependency plot for vehicle_manoeuvre (rural).

As with urban contexts, vehicle maneuvers played a major role in shaping severity (Fig 12). Notably, maneuvers such as going ahead on a bend and overtaking on the nearside are more frequently associated with fatal outcomes in the model predictions. These maneuvers inherently involve judgment under uncertainty, often at high speeds and on roads with limited visibility or insufficient signage. The elevated risk of nearside overtaking is especially problematic, as it contradicts established guidance such as Rule 268 of the UK Highway Code, which explicitly discourages overtaking on the left except in clearly defined circumstances [45]. These findings reinforce the need for driver education, signage enforcement and infrastructure adjustments, such as dedicated overtaking lanes or shoulder widening, to mitigate maneuver-related risks in rural settings. This aligns with findings from other safety-critical environments, where enhancing individual hazard recognition through knowledge-based training has shown measurable safety gains [46].

**Fig 13:**
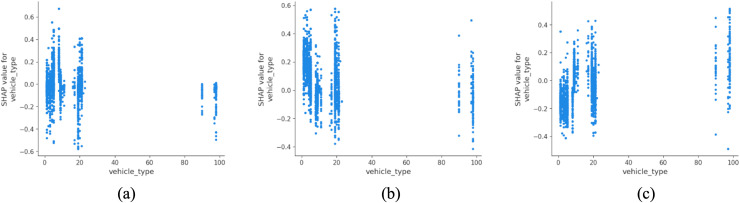
SHAP partial dependency plot for vehicle_type (rural).

Fig 13 shows that vehicle type again influences severity levels, with buses and coaches (type 11) demonstrating higher susceptibility to fatal outcomes. The increased passenger load, large vehicle mass and reduced maneuverability of these vehicles contribute to greater impact force and complexity during collision events. These results are consistent with recent analyses by Feng et al. (2016), who found that buses in rural areas exhibit elevated crash severity, especially in multi-vehicle incidents [47]. For rural sustainability, this emphasizes the need to balance public transport provision with infrastructure safety enhancements, particularly on narrow or high-speed corridors.

**Fig 14:**
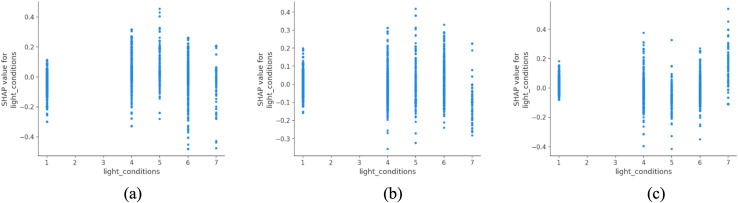
SHAP partial dependency plot for light_conditions (rural).

Lighting conditions, as shown in Fig 14, are strongly associated with differences in predicted accident severity in rural areas. Accidents occurring during daylight are more frequently associated with lower-severity outcomes, whereas crashes in unlit darkness are more often linked to fatal outcomes in the model predictions. The comparatively weaker association observed under artificial lighting conditions may reflect factors such as uneven lighting coverage, road geometry, traffic composition, or changes in driver behavior, rather than a direct protective effect of illumination alone. These patterns are consistent with findings reported by Uttley et al. (2020) and other rural lighting studies, which emphasize the challenges of ensuring safe night-time mobility in low-density or remote regions [48]. In this context, investments in low-energy and smart lighting systems should be viewed as part of a broader, integrated approach to rural infrastructure modernization, supporting both safety improvement and environmental efficiency.

### 5.4 Urban-rural comparison of risk factors

Table 4 presents the top ten most influential factors affecting accident severity across urban and rural settings. While certain predictors such as day of the week, vehicle maneuver, vehicle type and road surface conditions consistently rank among the top contributors in both contexts, notable divergences emerge, reflecting underlying spatial, infrastructural and behavioral differences between urban and rural transport environments.

**Table 4:** Comparison of key features for urban and rural.

	Urban	Rural
1	day_of_week	speed_limit
2	vehicle_manoeuvre	day_of_week
3	vehicle_type	vehicle_manoeuvre
4	junction_detail	vehicle_type
5	is_dst	light_conditions
6	road_surface_conditions	road_surface_conditions
7	junction_location	is_dst
8	light_conditions	road_type
9	speed_limit	junction_detail
10	pedestrian_crossing_human_contral	vehicle_leaving_carriageway

One of the most striking contrasts lies in the role of speed limit. In rural areas, speed limit ranks as the most critical factor, whereas it falls to ninth place in urban areas. This disparity underscores the structural centrality of speed control in rural safety management. Rural roads typically support higher design speeds, are subject to lower enforcement intensity, and often traverse areas with limited passive safety infrastructure. Consequently, even small increases in speed can drastically amplify crash severity. Conversely, in urban settings, where traffic congestion, signalization and pedestrian density naturally reduce effective driving speeds, posted speed limits play a more peripheral role, overshadowed by more immediate situational factors like maneuvering and junction complexity.

A similar asymmetry is observed in the variable junction detail, which ranks fourth in urban areas but only ninth in rural areas. This difference can be attributed to the higher density and complexity of intersections in urban contexts, where roundabouts, signalized junctions and pedestrian crossings concentrate conflict points within short spatial intervals. In such environments, minor infrastructural deficiencies or behavioral lapses can rapidly escalate into severe collisions. In contrast, rural roads tend to feature fewer junctions, often of simpler design, and lower overall traffic interaction.

Another noteworthy contrast arises from light conditions, which hold more weight in rural areas (ranked 5th) than in urban ones (ranked 8th). Urban areas generally benefit from denser and more uniform street lighting, while rural roads often lack adequate illumination, especially in peripheral or agricultural zones. The amplifying effect of darkness without lighting on crash severity in rural areas highlights a structural equity issue: rural residents face a disproportionately higher night-time traffic risk, which calls for targeted investment in lighting infrastructure as part of sustainable rural transport policy.

Additionally, the variable daylight-saving time (is_dst) appears in the top five for both urban and rural settings but is more impactful in urban areas, where it may better correlate with traffic demand shifts and driver fatigue patterns linked to social schedules. This suggests that temporal policy tools may have greater leverage in urban environments shaped by circadian mobility cycles.

Notably, pedestrian crossing controls emerge in the urban top ten but are absent from the rural ranking. This reflects the distinct modal mix in cities, where high pedestrian activity introduces more conflict with vehicles, especially in areas lacking signalized crossings or physical barriers. The absence of this variable in rural zones further emphasizes that urban traffic systems are inherently multi-modal, requiring a fundamentally different safety logic than rural systems, which remain predominantly vehicle centric.

### 5.5 Policy implications

The findings of this study highlight the necessity for spatially differentiated, evidence-based policy interventions that reflect the heterogeneous nature of road safety risks. Similar conclusions were drawn by recent urban safety research, which demonstrated that machine learning can be used not only to predict injury risk but also to guide context-specific interventions in line with urban governance and resilience objectives [49]. Uniform strategies are unlikely to produce equitable or sustainable outcomes, particularly when underlying risk factors diverge significantly between urban and rural settings. Leveraging interpretable machine learning models with disaggregated local data enables more precise, context-sensitive policy design, advancing the goals of resilience, safety and operational efficiency in regional transport systems.

In urban environments, both behavioral and infrastructural factors call for targeted measures. Notably, right-turn maneuvers emerged as a key contributor to accident severity, underscoring the need for intersection-level redesigns. Urban planners should consider implementing protected signal phases, dedicated turning lanes and physical channelization at high-risk junctions to mitigate conflicts involving oncoming traffic and vulnerable road users. These improvements not only enhance immediate safety but also facilitate multimodal integration – an essential pillar of sustainable urban mobility.

The temporal clustering of severe crashes during weekend transitions further suggests that time-sensitive enforcement and public awareness campaigns could address surges in high-risk behavior. Moreover, the involvement of large vehicles (e.g., buses and freight carriers) in high-severity accidents calls for time- and route-based operational restrictions during periods of peak pedestrian and cyclist exposure. Municipalities may also deploy smart logistics tools to balance commercial efficiency with urban safety imperatives. The diminished safety advantage during non-DST periods points to lighting quality as a critical concern. Expanding energy-efficient public lighting, especially in low-visibility corridors, combined with stricter nighttime visibility standards can improve safety without increasing energy demand.

In rural areas, distinct challenges are shaped by high-speed travel, sparse infrastructure, and lighting deficits. The strong association between high-speed zones and fatal crashes calls for the implementation of context-aware speed management, including adaptive speed limits below 60 km/h in high-risk segments and the installation of passive control devices such as rumble strips and road edge reflectors. Lighting again proves critical: smart solar-powered lighting at bends, junctions, and crossings should be prioritized in rural infrastructure upgrades to enhance both safety and energy equity. Overtaking-related hazards, particularly illegal nearside maneuvers, also warrant urgent attention. Reinforced regulatory signage, targeted driver education and monitoring technologies (e.g., in-vehicle alerts or UAV-based enforcement) can help curb such behavior. In extreme-risk zones, physical design interventions such as overtaking-restricted lanes or tactile warning surfaces may be necessary to ensure compliance.

More broadly, this study illustrates the utility of explainable AI tools such as SHAP in supporting anticipatory, spatially adaptive transport governance. By moving beyond retrospective crash statistics to reveal systemic risk factors embedded in environmental, vehicular and behavioral variables, transport authorities can prioritize resources more effectively. Importantly, the proposed framework relies exclusively on publicly available administrative accident data that are routinely collected by transport authorities, enhancing its scalability and transferability across regions without reliance on proprietary data sources or additional data collection mechanisms. This approach aligns with the principles of intelligent urban transition by enabling proactive, localized and transparent policy action, ultimately contributing to the development of low-carbon, inclusive and resilient mobility systems.

## 6. Conclusions

This study proposes a spatially disaggregated, interpretable machine learning framework to examine the determinants of traffic accident severity in urban and rural areas of Kent, UK. By modeling these environments separately, the analysis reveals how risk structures differ substantially across spatial contexts, challenging the validity of one-size-fits-all approaches in road safety governance.

Leveraging three years of disaggregated crash data and five machine learning models, the RF algorithm demonstrated superior predictive performance and was selected for explainability analysis using SHAP. This allowed the extraction of con-text-specific insights that go beyond black-box predictions, empowering policymakers to understand not only what the risk factors are, but how they operate across space and time.

Findings indicate that high-impact predictors such as vehicle maneuver, vehicle type and day of the week affect accident severity in both settings. However, spatial divergences also emerged: urban severity was associated with intersection complexity and daylight savings periods, while rural crashes were more sensitive to speed limits and lighting conditions. These insights point to the value of context-aware interventions, such as adaptive speed management in rural corridors and intersection-specific redesigns in urban areas. Rather than relying on retrospective crash statistics, the integration of explainable machine learning allows local authorities to identify latent risk patterns based on real-world behavioral, infrastructural and temporal signals. For example, SHAP-based analyses can inform dynamic signal phasing at high-risk junctions, prioritize lighting upgrades in poorly lit segments, or guide time-based restrictions for heavy vehicle movement.

From a local governance perspective, this approach enhances the precision of transport safety budgeting by enabling regional and municipal planners to allocate resources toward high-impact locations with clearer empirical justification. Similar to findings in recent literature, the use of interpretable machine learning models to support black spot identification has proven effective in informing spatially targeted, cost-efficient road safety interventions [50]. It also illustrates how anticipatory safety planning could be supported at the local level, where interventions are informed not only by past incidents but also by emerging spatial configurations of risk identified through interpretable modeling. In this way, the proposed framework demonstrates the potential for data-informed governance to move beyond reactive responses toward more proactive and spatially responsive safety planning in similar regional contexts.

## Supporting information

A1 Table Descriptive statistics (urban). (DOCX)

A2 Table Descriptive statistics (rural). (DOCX)

B1 Table Per-class precision, recall, and F1-scores for the Random Forest model (urban). (DOCX)

B2 Table Per-class precision, recall, and F1-scores for the Random Forest model (rural). (DOCX)

B1 Fig Normalized confusion matrix for the Random Forest model (urban accidents). (DOCX)

B2 Fig Normalized confusion matrix for the Random Forest model (rural accidents). (DOCX)

## Abbreviations

ADASYN: Adaptive Synthetic Sampling
CNN: convolutional neural network
DST: daylight saving time
DT: Decision Tree
GBT: Gradient Boosted Trees
LR: Logistic Regression
RF: Random Forest
SHAP: : Shapley Additive exPlanations
SMOTE: Synthetic Minority Oversampling Technique
SVC: Support Vector Classifier
SVM: Support Vector Machine
