# Supplementary material for: Resilient road safety modeling through spatially disaggregated explainable AI
Source: PLoS One. 2026 Apr 24;21(4):e0344380. doi: 10.1371/journal.pone.0344380 (PMC13108897; doi:10.1371/journal.pone.0344380)
Supplement: A1 Table — (DOCX) [file pone.0344380.s001.docx]

Table A1. Descriptive statistics (urban).

| Variable | Code | Top categories | Frequency/Mean |
| --- | --- | --- | --- |
| accident_severity | 3 | Slight | 10652 |
|  | 2 | Serious | 2358 |
|  | 1 | Fatal | 236 |
| day_of_week | 6 | Friday | 2169 |
|  | 5 | Thursday | 2125 |
|  | 4 | Wednesday | 1942 |
| time |  | 17：00 | 142 |
|  |  | 15：00 | 101 |
|  |  | 12：00 | 92 |
| road_type | 6 | Single carriageway | 7353 |
|  | 3 | Dual carriageway | 4111 |
|  | 1 | Roundabout | 929 |
| speed_limit |  | 70 | 3469 |
|  |  | 60 | 3370 |
|  |  | 30 | 3348 |
| junction_detail | 0 | Not at junction or within 20 metres | 8834 |
|  | 3 | T or staggered junction | 1965 |
|  | 1 | Roundabout | 1039 |
| junction_control | -1 | Data missing or out of range | 8950 |
|  | 4 | Give way or uncontrolled | 3725 |
|  | 2 | Auto traffic signal | 271 |
| pedestrian_crossing_human_control | 0 | None within 50 metres | 13128 |
|  | 2 | Control by other authorised person | 78 |
| pedestrian_crossing_physical_facilities | 0 | No physical crossing facilities within 50 metres | 12863 |
|  | 4 | Pelican, puffin, toucan or similar non-junction pedestrian light crossing | 115 |
| light_conditions | 1 | Daylight | 9824 |
|  | 4 | Darkness - lights lit | 1530 |
|  | 6 | Darkness - no lighting | 1384 |
| weather_conditions | 1 | Fine no high winds | 10754 |
|  | 2 | Raining no high winds | 1309 |
|  | 9 | Unknown | 288 |
| road_surface_conditions | 1 | Dry | 9569 |
|  | 2 | Wet or damp | 3174 |
|  | 4 | Frost or ice | 318 |
| special_conditions_at_site | 0 | None | 12818 |
|  | 4 | Roadworks | 187 |
| carriageway_hazards | 0 | None | 12826 |
|  | 2 | Other object on road | 177 |
| vehicle_type | 9 | Car | 9882 |
|  | 19 | Van / Goods 3.5 tonnes mgw or under | 857 |
|  | 1 | Pedal cycle | 489 |
| vehicle_manoeuvre | 18 | Going ahead other | 6694 |
|  | 4 | Slowing or stopping | 1306 |
|  | 3 | Waiting to go - held up | 930 |
| vehicle_location_restricted_lane | 0 | On main c'way - not in restricted lane | 13005 |
|  | 6 | On lay-by or hard shoulder | 77 |
| junction_location | 0 | Not at or within 20 metres of junction | 8834 |
|  | 1 | Approaching junction or waiting/parked at junction approach | 2285 |
|  | 8 | Mid Junction - on roundabout or on main road | 641 |
| vehicle_leaving_carriageway | 0 | Did not leave carriageway | 11250 |
|  | 1 | Nearside | 1018 |
|  | 7 | Offside | 462 |
| vehicle_left_hand_drive | 1 | No | 13190 |
|  | 2 | Yes | 55 |
| sex_of_driver | 1 | Male | 8732 |
|  | 2 | Female | 3884 |
|  | 3 | Not known | 630 |
| age_of_driver |  |  | Min.= -1.0 |
|  |  |  | Median=37.0 |
|  |  |  | Mean=38.0 |
|  |  |  | Max.=98.0 |
|  |  |  | SD=19.5 |
| age_band_of_driver | 6 | 26 - 35 | 2692 |
|  | 7 | 36 - 45 | 2514 |
|  | 8 | 46 - 55 | 2159 |
| engine_capacity_cc |  |  | Min.= -1.0 |
|  |  |  | Median=1422.0 |
|  |  |  | Mean=1472.5 |
|  |  |  | Max.=99999.0 |
|  |  |  | SD=1780.9 |
| propulsion_code | 1 | Petrol | 5676 |
|  | 2 | Heavy oil | 4679 |
| age_of_vehicle |  |  | Min.= -1 |
|  |  |  | Median=5.0 |
|  |  |  | Mean=6.2 |
|  |  |  | Max.=61.0 |
|  |  |  | SD=6.2 |
