# Supplementary material for: Resilient road safety modeling through spatially disaggregated explainable AI
Source: PLoS One. 2026 Apr 24;21(4):e0344380. doi: 10.1371/journal.pone.0344380 (PMC13108897; doi:10.1371/journal.pone.0344380)
Supplement: A2 Table — (DOCX) [file pone.0344380.s002.docx]

Table A2. Descriptive statistics (rural).

| Variable | Code | Top categories | Frequency/Mean |
| --- | --- | --- | --- |
| accident_severity | 3 | Slight | 11585 |
|  | 2 | Serious | 1958 |
|  | 1 | Fatal | 73 |
| day_of_week | 6 | Friday | 2326 |
|  | 5 | Thursday | 2075 |
|  | 3 | Tuesday | 2021 |
| time |  | 18：00 | 139 |
|  |  | 15：30 | 122 |
|  |  | 17：30 | 118 |
| road_type | 6 | Single carriageway | 10128 |
|  | 3 | Dual carriageway | 1587 |
|  | 1 | Roundabout | 758 |
| speed_limit |  | 30 | 11559 |
|  |  | 40 | 977 |
|  |  | 70 | 369 |
| junction_detail | 0 | Not at junction or within 20 metres | 6601 |
|  | 3 | T or staggered junction | 3892 |
|  | 6 | Crossroads | 960 |
| junction_control | -1 | Data missing or out of range | 6789 |
|  | 4 | Give way or uncontrolled | 5401 |
|  | 2 | Auto traffic signal | 1039 |
| pedestrian_crossing_human_control | 0 | None within 50 metres | 13348 |
|  | 2 | Control by other authorised person | 180 |
| pedestrian_crossing_physical_facilities | 0 | No physical crossing facilities within 50 metres | 11342 |
|  | 4 | Pelican, puffin, toucan or similar non-junction pedestrian light crossing | 911 |
|  | 5 | Pedestrian phase at traffic signal junction | 622 |
| light_conditions | 1 | Daylight | 10084 |
|  | 4 | Darkness - lights lit | 2978 |
|  | 7 | Darkness - lighting unknown | 279 |
| weather_conditions | 1 | Fine no high winds | 11372 |
|  | 2 | Raining no high winds | 1270 |
|  | 9 | Unknown | 297 |
| road_surface_conditions | 1 | Dry | 10612 |
|  | 2 | Wet or damp | 2780 |
|  | 4 | Frost or ice | 132 |
| special_conditions_at_site | 0 | None | 13346 |
|  | 4 | Roadworks | 84 |
| carriageway_hazards | 0 | None | 13358 |
|  | 2 | Other object on road | 110 |
| vehicle_type | 9 | Car | 10380 |
|  | 1 | Pedal cycle | 883 |
|  | 19 | Van / Goods 3.5 tonnes mgw or under | 581 |
| vehicle_manoeuvre | 18 | Going ahead other | 6402 |
|  | 2 | Parked | 1187 |
|  | 9 | Turning right | 1164 |
| vehicle_location_restricted_lane | 0 | On main c'way - not in restricted lane | 13196 |
|  | 9 | Footway (pavement) | 256 |
| junction_location | 0 | Not at or within 20 metres of junction | 6601 |
|  | 1 | Approaching junction or waiting/parked at junction approach | 3821 |
|  | 8 | Mid Junction - on roundabout or on main road | 1067 |
| vehicle_leaving_carriageway | 0 | Did not leave carriageway | 12754 |
|  | 1 | Nearside | 439 |
|  | 7 | Offside | 208 |
| vehicle_left_hand_drive | 1 | No | 13600 |
|  | 2 | Yes | 16 |
| sex_of_driver | 1 | Male | 8410 |
|  | 2 | Female | 4184 |
|  | 3 | Not known | 1022 |
| age_of_driver |  |  | Min.= -1 |
|  |  |  | Median=34 |
|  |  |  | Mean=36.4 |
|  |  |  | Max.=91 |
|  |  |  | SD=20.9 |
| age_band_of_driver | 6 | 26 - 35 | 2685 |
|  | 7 | 36 - 45 | 2430 |
|  | 8 | 46 - 55 | 1876 |
| engine_capacity_cc |  |  | Min.= -1 |
|  |  |  | Median=1398 |
|  |  |  | Mean=1357.8 |
|  |  |  | Max.=16120 |
|  |  |  | SD=1300.6 |
| propulsion_code | 1 | Petrol | 6232 |
|  | 2 | Heavy oil | 4309 |
| age_of_vehicle |  |  | Min.= -1 |
|  |  |  | Median=6 |
|  |  |  | Mean=6.5 |
|  |  |  | Max.=91 |
|  |  |  | SD=6.3 |
