# Supplementary material for: Resilient road safety modeling through spatially disaggregated explainable AI
Source: PLoS One. 2026 Apr 24;21(4):e0344380. doi: 10.1371/journal.pone.0344380 (PMC13108897; doi:10.1371/journal.pone.0344380)
Supplement: B1 Table — (DOCX) [file pone.0344380.s003.docx]

Table B1. Per-class precision, recall, and F1-scores for the Random Forest model (urban).

| **Class** | **Precision** | **Recall** | **F1-score** |
| --- | --- | --- | --- |
| Slight | 0.96 | 0.97 | 0.96 |
| Serious | 0.78 | 0.72 | 0.75 |
| Fatal | 0.60 | 0.35 | 0.44 |
