# Supplementary figures and images for: Resilient road safety modeling through spatially disaggregated explainable AI
Source: PLoS One. 2026 Apr 24;21(4):e0344380. doi: 10.1371/journal.pone.0344380 (PMC13108897; doi:10.1371/journal.pone.0344380)

Figure B1. Normalized confusion matrix for the Random Forest model (urban accidents).


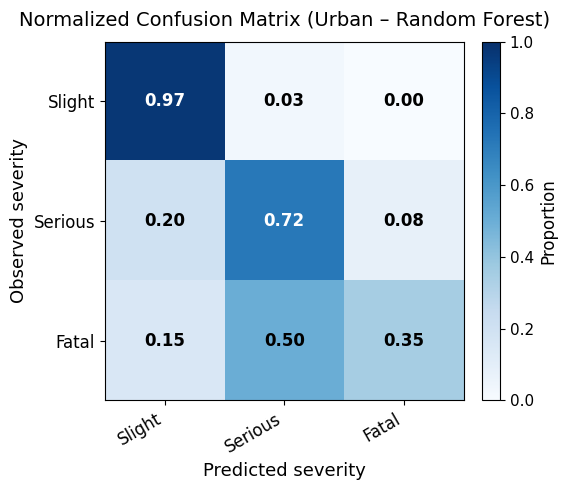

Supplement: B1 Fig — (DOCX) [file pone.0344380.s005.docx]

Figure B2. Normalized confusion matrix for the Random Forest model (rural accidents).


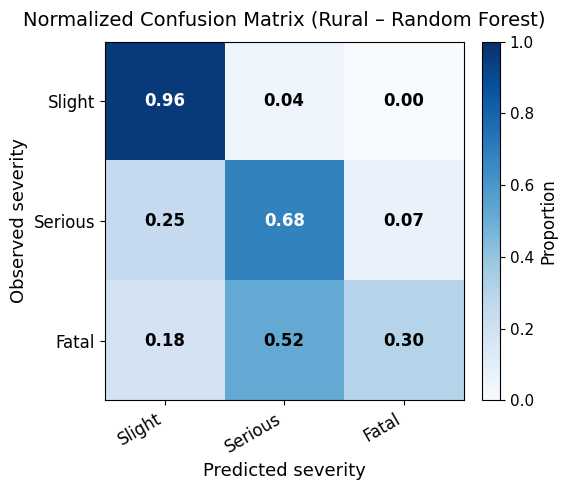

Supplement: B2 Fig — (DOCX) [file pone.0344380.s006.docx]
